# Significant anti-myeloma potential of green tea infusion and its component dihydromyricetin

**DOI:** 10.1097/MS9.0000000000005494

**Published:** 2026-08-06

**Authors:** Juan Xiao, Hongyu Zhu, Yanxiao Han, Chenliu Fan, Yuyan Chen, Xiaoli Liu, Chengyun Zheng, Yang Jiang

**Affiliations:** a Department of Hematology, The Second Qilu Hospital of Shandong University, Jinan, Shandong, China; b Institute of Biotherapy for Hematological Malignancy, Shandong University, Jinan, Shandong, China; c Shandong University-Karolinska Institute Collaboration Laboratory for Stem Cell Research, Jinan, Shandong, China; d Department of Hematology, Jinan Fourth People’s Hospital, Jinan, Shandong, China; e Department of Reproductive Medicine, The Second Qilu Hospital of Shandong University, Jinan, Shandong, China

**Keywords:** apoptosis, dihydromyricetin, green tea, multiple myeloma

## Abstract

Multiple myeloma (MM) is a fatal hematological malignancy characterized by overgrowth of monoclonal plasma cells. Although green tea exhibits well-documented antitumor properties, its specific effects against MM remain poorly characterized. This study aimed to investigate the anti-myeloma activity of infusions from four representative green teas and identify their effective components. We found that apoptosis of MM cells was induced by green tea infusions in a concentration-dependent manner, with Biluochun tea infusion showing the most potent efficacy. An animal study showed that Biluochun tea infusion significantly reduced MM tumor size, downregulated the proliferation marker Ki-67, and increased TUNEL-positive apoptotic cells in MM tumor tissues. Furthermore, mass spectrometry analysis identified dihydromyricetin (DMY) as a pivotal distinguishing component of Biluochun among the four tea infusions. As expected, DMY induced apoptosis in MM cells in a concentration-dependent manner, accompanied by decreased mitochondrial membrane potential and elevated expression of cleaved caspase-3 and caspase-8. These findings indicate that DMY elicits anti-myeloma effects through activation of both the intrinsic mitochondrial and extrinsic apoptotic pathways. Collectively, this study provides preliminary preclinical evidence supporting the anti-myeloma activity of DMY in cell and xenograft mouse models, which lays a foundation for subsequent translational research exploring green tea-derived compounds against MM; further multilayered validation is required to confirm its clinical value.

## Introduction

Multiple myeloma (MM) is a malignant hematological disorder characterized by clonal malignant plasma cell proliferation and accumulation in the bone marrow, accounting for approximately 10% of all hematological malignancies^[^1^]^. As the second most prevalent hematological malignancy in developed countries, MM primarily occurs in the elderly population and presents substantial clinical and socioeconomic burdens. The typical clinical manifestations of MM include hypercalcemia, renal failure, anemia, or osteolytic lesions, which are responsible for most disease-associated morbidities. Detailed diagnostic tests and timely therapeutic intervention are essential to prevent the severe consequences associated with these symptoms and improve the overall prognosis of MM patients^[^2^]^. The clinical application of immunomodulatory drugs, proteasome inhibitors, and targeted monoclonal antibodies has greatly advanced MM treatment and significantly prolonged patient survival in recent years. However, despite these therapeutic improvements, MM remains an incurable hematological malignancy to date. Furthermore, some MM patients experience fatal adverse events and systemic complications arising from long-term clinical treatment, further worsening clinical outcomes^[^3^]^. Moreover, the ubiquitous development of primary and acquired drug resistance frequently leads to disease relapse and treatment failure, constituting a major obstacle to durable MM therapy. Given these persistent clinical challenges, it is imperative and clinically meaningful to continuously explore novel therapeutic agents and alternative strategies for the improved management of MM.

HIGHLIGHTS

- Green tea infusions induced apoptosis in multiple myeloma (MM) cells in a concentration-dependent manner.
- Dihydromyricetin (DMY), an abundant component of Biluochun, has anti-MM activity.
- DMY induced apoptosis in MM cells through both mitochondrial and non-mitochondrial pathways.
- Green teas and their chemical components may enhance MM treatments.

Green tea, derived from the fresh leaves of the tea plant (*Camellia sinensis L.*), represents one of the most widely consumed traditional beverages worldwide, particularly popular in Southeast Asian countries. Accumulating evidence from pharmacological studies has demonstrated that green tea possesses extensive health-beneficial properties, which are largely mediated by the modulation of multiple intracellular signaling cascades closely associated with cell proliferation, apoptosis, and inflammation^[^4^]^. Tea polyphenols are the predominant bioactive phytochemicals in green tea leaves, accounting for 15%–35% of the total dry weight of tea. This complex group of phenolic compounds primarily consists of catechins, flavonoids, anthocyanins, and phenolic acids. Among these constituents, catechins serve as the principal functional fraction, constituting approximately 70% of the total tea polyphenol content^[^5^]^. Notably, epigallocatechin-3-gallate (EGCG) is the most abundant and biologically active monomer within tea catechins and has been well documented to exhibit important antitumor activity across diverse malignancies^[^6^]^. Accumulating preclinical evidence has verified that EGCG can effectively inhibit adrenal, bladder, breast, cervical, colorectal, esophageal, stomach, liver, lung, oral, ovarian, pancreatic, prostate, and skin cancer cells *in vitro*^[^7^]^. Consistent with these findings, our previous data highlight that EGCG has potent anti-MM activity *in vitro* and *in vivo*^[^8^]^, further confirming its promising therapeutic potential against MM. However, the specific roles of green teas, especially those originating from different sources, as well as the potential anti-MM functions and molecular mechanisms of other core active ingredients apart from EGCG, remain largely unclear. This knowledge gap restricts the comprehensive understanding of the medicinal value of green tea and limits the discovery of novel anti-myeloma natural compounds.

In the present study, we conducted a comparative analysis of the anti-MM activity of tea infusions from four representative green teas sourced from different producing regions. Mass spectrometry-based metabolomic analysis was additionally performed to screen for and characterize differential bioactive components among the four green tea infusions. The aim of this study was to screen green tea infusions for in vitro and in vivo anti-MM activity, to identify their characteristic bioactive components, and to uncover the underlying apoptotic molecular mechanisms; the translational potential of these compounds will require additional preclinical and clinical verification in future work. All experimental procedures in this study were conducted in strict accordance with the TITAN Guidelines 2025^[^9^]^.

## Materials and methods

### Cell lines and culture

The human MM cell lines RPMI8226 (CCL-155™) and U266 (TIB-196™) were commercially obtained from the American Type Culture Collection (Manassas, VA, USA). Both cell lines were routinely maintained in Roswell Park Memorial Institute (RPMI) 1640 medium (Gibco, Grand Island, NY, USA) containing 10% fetal bovine serum (10 100 147, Gibco) and 1% penicillin-streptomycin (15 140 122, Gibco). All cells were incubated in a humidified incubator under standardized culture conditions of 37 °C and 5% carbon dioxide (CO₂). The culture medium was refreshed every 2 days, and cells in the logarithmic growth phase were collected for subsequent experimental studies. All cell functional experiments described below were repeated for a minimum of three independent biological replicates or three technical replicates to guarantee experimental reproducibility.

### Tea and tea decoction preparation

Four typical varieties of alpine green tea originating from their core producing areas were selected and collected for this study, including Maofeng tea from Emei Mountain in Sichuan Province, Biluochun tea from Dongting Mountain in Jiangsu Province, West Lake Longjing tea from Hangzhou City in Zhejiang Province, and Laoshan tea from Laoshan Mountain in Shandong Province. Subsequently, 3.0 g of each green tea sample was precisely weighed using an electronic balance and transferred into separate 50 mL centrifuge tubes. A total volume of 30 mL of prefiltered pure water was added to each tube, followed by brewing with boiling water for 6 minutes. After brewing, all tea infusions were centrifuged at 4000*g* at 4 °C for 10 minutes. The clarified supernatant from each sample was collected, filtered through a 0.22 µm microporous membrane, transferred into sterilized tubes, and preserved in a −80 °C freezer for subsequent experiments.

### Detection of apoptotic cells using flow cytometry

The MM cell lines RPMI 8226 and U266 in the logarithmic growth phase were seeded into 24-well plates at a density of 2 × 10^5^ cells/mL. The MM cells were treated separately with four types of green tea infusions at graded concentrations (0, 0.0625, 0.125, and 0.25 g/L) or dihydromyricetin (DMY; 0, 20, 40, and 80 μM) for 24 h. Untreated cells cultured in complete medium were defined as the blank control, and cells treated with an equal volume of solvent (sterile pure water for tea infusions; DMSO for DMY) were uniformly defined as the vehicle control. After culture, cell suspensions were collected and centrifuged. After removing the supernatant, the cell pellets were washed twice with cold phosphate-buffered saline (PBS). Before adding 2 μL of Annexin V-FITC and 2 μL of propidium iodide (PI), the cells were resuspended in 200 μL of buffer. The staining mixture was incubated at 4 °C in the dark for 15 min. After incubation, 400 μL of PBS was added to each sample tube. Finally, flow cytometry (Beckman, Miami, FL, USA) was used to detect cell apoptosis. The proportions of early apoptotic cells (Annexin V⁺PI^−^) and late apoptotic or necrotic cells (Annexin V⁺PI⁺) were statistically analyzed, and the total apoptosis rate of each group was calculated accordingly.

### Establishment of an MM xenograft tumor mouse model

Animal experiments were approved by the Animal Ethics Committee of the Second Qilu Hospital of Shandong University (KYLL-2020(KJ)A-0140). Twelve SPF-grade female NCG mice aged 6–8 weeks were purchased from GemPharmatech Co., Ltd. (Nanjing, China). All experimental mice were accompanied by complete certificates of origin and qualification and had not undergone any prior experimental interventions. Before formal modeling, all mice (4 mice per cage) were acclimated for 7 days under SPF conditions (22 ± 2 °C, 50 ± 5% humidity, 12 h light–dark cycle) with free access to sterilized rodent feed and filtered water. All animal grouping, administration, and sampling procedures were standardized to ensure full reproducibility: the collected RPMI-8226 cells (1 × 10^7^ cells/mouse) were injected subcutaneously into the right forelimb of each mouse. After tumor formation, the maximum diameter (a) and minimum diameter (b) of the tumors were measured with a vernier caliper every 3 days, and the tumor volume was calculated according to the following formula: V = 1/2 × ab^2^. When the tumor volume reached approximately 100 mm^3[^10^]^, mice were randomly divided into three groups via the GraphPad Prism 8.0 random number generator (*n* = 4 per group): a vehicle control group given daily intragastric administration of an equal-volume of sterile water, a Maofeng group administrated with 4 mg of Maofeng tea extract, and a Biluochun group with 4 mg of Biluochun tea extract via intragastric gavage once daily at a fixed time, with a uniform injection volume of 100 μL per mouse. The tumor diameter was measured using a vernier caliper every 3 days, and the volume was calculated. The humane experimental termination criteria were strictly formulated: the experiment was terminated once the unilateral maximum tumor diameter exceeded 1.5 cm in any direction or the mice presented obvious disorders of crawling, drinking, or eating. A single-blind experimental design was implemented: the operators responsible for intragastric administration knew the group assignments, whereas the staff engaged in tumor measurement and subsequent statistical analysis of the data remained unaware of group assignments until all experimental data collection was completed. Once mice in the control group reached the experimental endpoint, all mice in each group were humanely euthanized via cervical dislocation, and the tumor tissues were completely isolated, photographed, compared, recorded, and fixed in 4% paraformaldehyde. This work has been reported in line with the ARRIVE criteria^[^11^]^.

### Immunohistochemical staining for Ki-67 and TUNEL

The paraffin sections of tumors were prepared following an overnight fixation in formalin. For further histological assessment, Ki-67 immunohistochemical staining and TUNEL apoptosis staining were sequentially performed on the prepared paraffin sections using the Ki-67 Antibody Kit (GB111141, Servicebio, Wuhan, China) and the TUNEL Cell Apoptosis Detection Kit (G1507, Servicebio), respectively. All staining procedures were strictly carried out in accordance with the detailed official experimental protocols provided with each kit. After staining was completed, all tissue sections were scanned with a digital slide scanner to obtain high-resolution panoramic histological images. The areas of positive expression of the target markers were quantitatively analyzed using Image-Pro Plus software (Version X; Media Cybernetics, Silver Spring, MD, USA). Immunohistochemical quantification was performed on three independent tumor tissue sections per mouse, with three random visual fields captured per section for statistical analysis.

### Mass spectrometric analysis of green tea components

A 200 μL aliquot of supernatant from each of the four tea infusions was transferred into individual 1.5 mL EP tubes. Five independent biological replicates of each tea sample were prepared for mass spectrometric analysis to reduce detection bias. All samples were subjected to component identification via high-performance liquid chromatography-tandem mass spectrometry (HPLC-MS/MS), and the entire instrumental detection process was conducted by Shanghai Biotree Biotech Co., Ltd. (Shanghai, China). Prior to formal testing, sample pretreatment, including centrifugation and membrane filtration, was carried out to remove impurities. After mass spectrometry data collection, raw spectra were first calibrated and denoised, followed by peak matching, comparison with standard substances, and qualitative identification of chemical components. The relative content of each identified component was further quantified through peak area normalization. Based on the finalized mass spectrometric data, we compared the compositional discrepancies among the four green tea samples and further clarified the unique chemical components and content differences between Biluochun tea and the other three green tea varieties.

### Detection of mitochondrial membrane potential

RPMI 8226 or U266 cells were seeded into 24-well plates and treated for 24 h with Biluochun tea infusion at concentrations of 0 and 0.25 g/L, as well as with a concentration gradient of DMY at 0, 20, 40, and 80 μM for 24 h respectively. The JC-1 mitochondrial membrane potential detection kit (Cat. No. 40706ES60, YEASEN Biotechnology Co., Ltd., Shanghai, China) was used in this experiment. Following the manufacturer's instructions, we prepared the JC-1 working solution and 1× incubation buffer in advance. After discarding the culture medium, the treated cells were rinsed thoroughly with precooled PBS. Then, 500 μL of prepared JC-1 working solution was added and mixed thoroughly with the cells, and the mixture was incubated at room temperature for 20 min, protected from light, to complete fluorescent staining. Subsequently, the cells were washed twice with 1 mL of 1× incubation buffer and resuspended in 500 μL of 1× incubation buffer. Flow cytometry was used to analyze changes in mitochondrial membrane potential by recording green monomer and red aggregate fluorescence signals, with the red/green fluorescence intensity ratio and the percentage of JC-1 monomer-positive cells quantified to evaluate mitochondrial depolarization, an early apoptotic marker.

### Western blot analysis

Total cellular protein was extracted from RPMI 8226 or U266 cells using radioimmunoprecipitation assay lysis buffer (R0010, Solarbio) supplemented with protease inhibitors. Subsequently, the protein concentrations of each sample were determined using a BCA Protein Quantification Kit (20201ES76, YEASEN). Equal amounts of denatured protein samples were separated by sodium dodecyl sulfate–polyacrylamide gel electrophoresis and then transferred onto polyvinylidene fluoride membranes (ISEQ00010, Millipore, MA, USA). After blocking with 5% BSA, the membrane was incubated overnight at 4 °C with primary antibodies against β-actin (3700S, CST), caspase-3 (9662S, CST), and caspase-8 (9746T, CST) at a 1:1000 dilution. After thorough washing, the membrane was incubated with a horseradish peroxidase–conjugated secondary antibody [HRP-conjugated goat anti-rabbit IgG (H + L), AS014, ABclonal] at room temperature for 1 h. A fresh chemiluminescent substrate working solution was prepared by mixing equal volumes of solutions A and B (RM00021P, ABclonal) according to the instructions and was applied to fully cover the membrane. Finally, protein band signals were captured and visualized using a Tanon 5200 chemiluminescence imaging system (Shanghai, China). The relative expression levels of target proteins were normalized to β-actin, which served as the internal reference protein. All Western blot experiments were performed in three independent biological replicates.

### Statistical analysis

Statistical analyses were performed using GraphPad Prism 8.0 (La Jolla, CA, USA). All data are presented as means ± standard error of the mean (SEM). The normality of continuous data was assessed using the Shapiro–Wilk test. For normally distributed data, one-way ANOVA was used to examine differences among multiple groups, whereas Student’s *t*-test was used to compare two groups. For non-normally distributed data, the nonparametric Kruskal–Wallis test was used. A *P*-value < 0.05 was considered statistically significant.

## Results

### Four green tea infusion induced RPMI8226 cell apoptosis in a concentration-dependent manner

**Figure 1.**
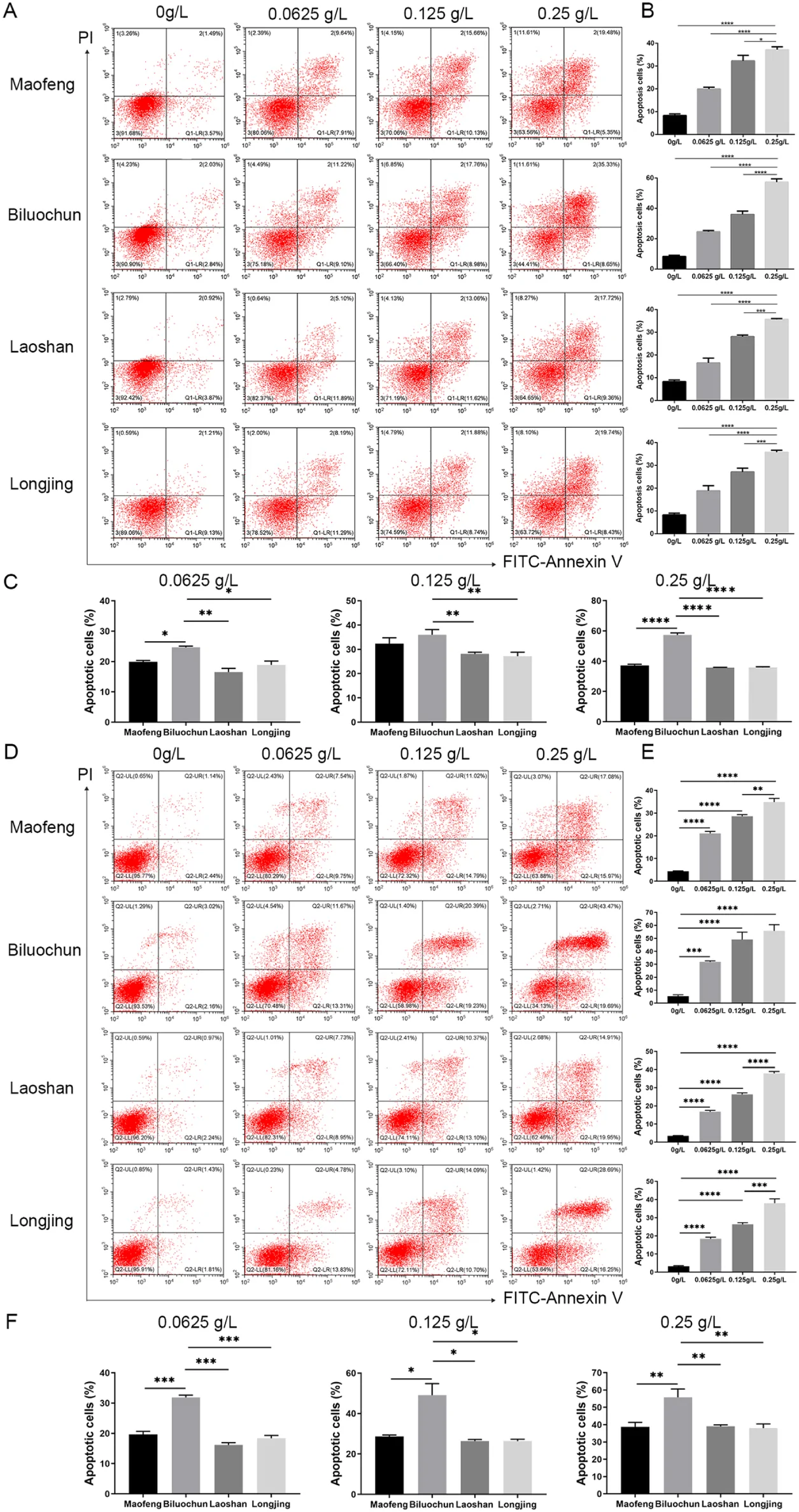
Apoptosis induction in MM cells treated with four green tea infusions. (A) Representative flow cytometry plots of Annexin V/PI staining in RPMI 8226 cells treated with graded concentrations of the four green tea infusions for 24 hours. (B) Quantitative analysis of the percentages of apoptotic (both PI^+^ and Annexin V^+^) RPMI 8226 cells after treatment with the indicated concentrations of the four infusions for 24 hours. (C) Comparative analysis of the percentages of apoptotic-positive RPMI 8226 cells among the four infusions at the same treatment concentration. (D) Representative flow cytometry plots of Annexin V-FITC/PI staining in U266 cells after 24 h of treatment with the four green tea infusions at graded concentrations. (E) Quantitative analysis of the total apoptotic rate of U266 cells after treatment with different green tea infusions. (F) Comparative analysis of the percentages of apoptotic U266 cells among the four green tea infusions at the same treatment concentration. All blank groups are defined as medium-only controls; solvent-treated groups are uniformly designated as vehicle controls in all panels. *P < 0.05, **P < 0.01, ***P < 0.001, ****P < 0.0001.

To explore the potential apoptosis-inducing effects of four representative green tea varieties cultivated in their respective regions of origin in China on MM cells, infusions of Maofeng, Biluochun, Laoshan, and Longjing were prepared following the procedures outlined in the Materials and Methods section. The results showed that all four green tea infusions induced apoptosis in RPMI 8226 cells (Fig. 1A–C) and U266 cells (Fig. 1D–F) in a concentration-dependent manner. In particular, the Biluochun tea infusion exhibited more potent pro-apoptotic activity at all three experimental concentrations than the other three green tea varieties (Fig. 1C and F). Considering its consistent and superior anti-myeloma pro-apoptotic performance across graded concentrations, the Biluochun tea infusion was selected for further mechanistic exploration and subsequent functional validation experiments.

### Biluochun infusion reduced mitochondrial membrane potential of MM cells

**Figure 2.**
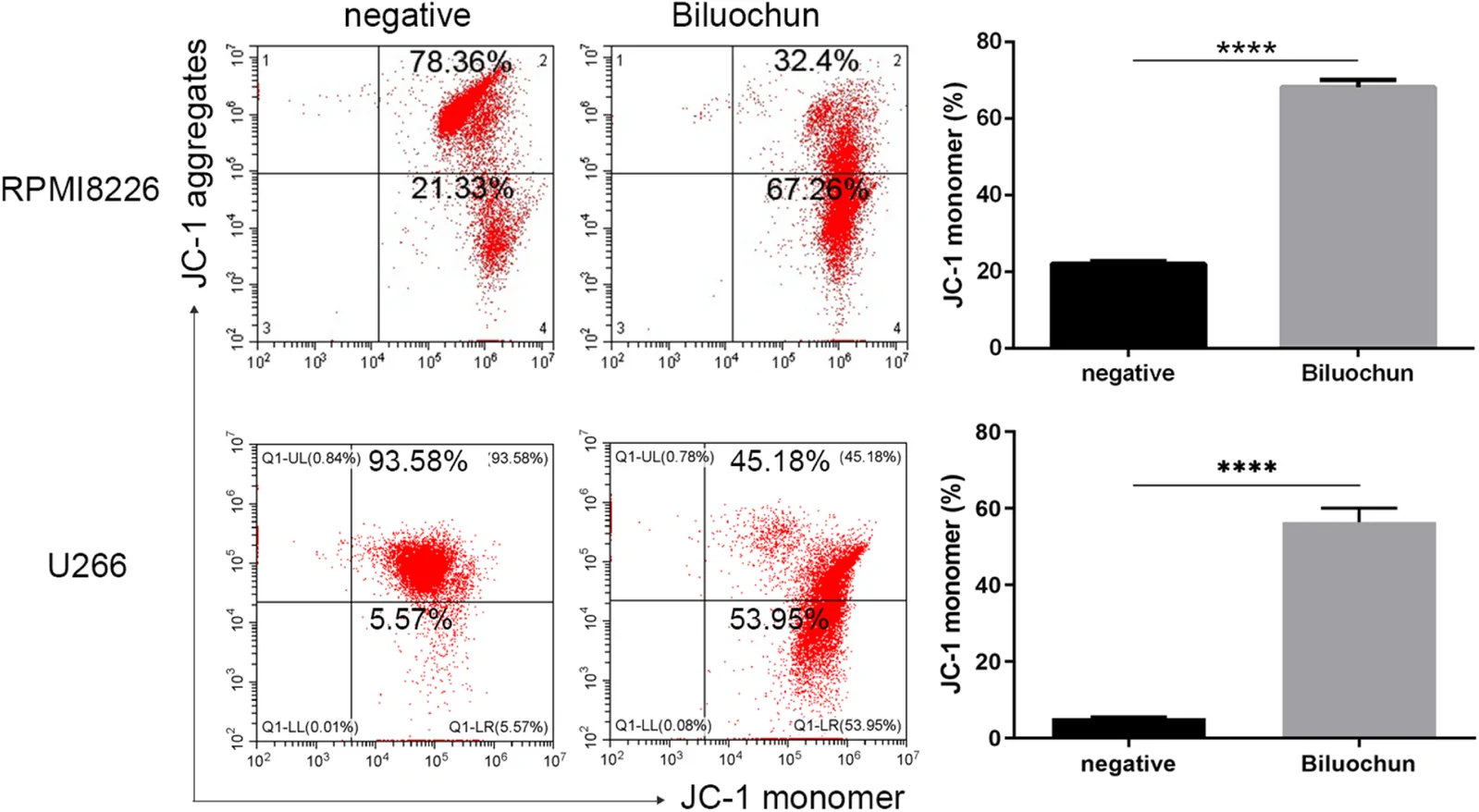
Alterations in MMP in MM cells following Biluochun infusion treatment. JC-1 staining combined with flow cytometry was used to evaluate changes in MMP in RPMI 8226 and U266 cells treated with 0.25 g/L Biluochun infusion for 24 h. The 0 g/L Biluochun group represents the vehicle control. Data are presented as the mean ± SEM of no fewer than three independent experiments. ****P < 0.0001.

To further elucidate the regulatory effect of Biluochun tea infusion on mitochondrial function during apoptosis in MM cells, the mitochondrial membrane potential (MMP) was analyzed. As shown in Figure 2, after treatment with Biluochun tea infusion for 24 h, the percentage of JC-1 monomer-positive RPMI 8226 cells increased from 21.33% to 67.26%, while that in U266 cells rose from 5.57% to 53.95%, both of which were significantly higher than in the corresponding vehicle control groups. The increased proportion of JC-1 monomers directly reflects a marked decline in mitochondrial membrane potential. These results confirm that Biluochun tea infusion prominently induces depolarization of the mitochondrial membrane potential in both myeloma cell lines, a typical early marker of apoptosis.

### Green tea infusions inhibited MM cell growth in vivo

**Figure 3.**
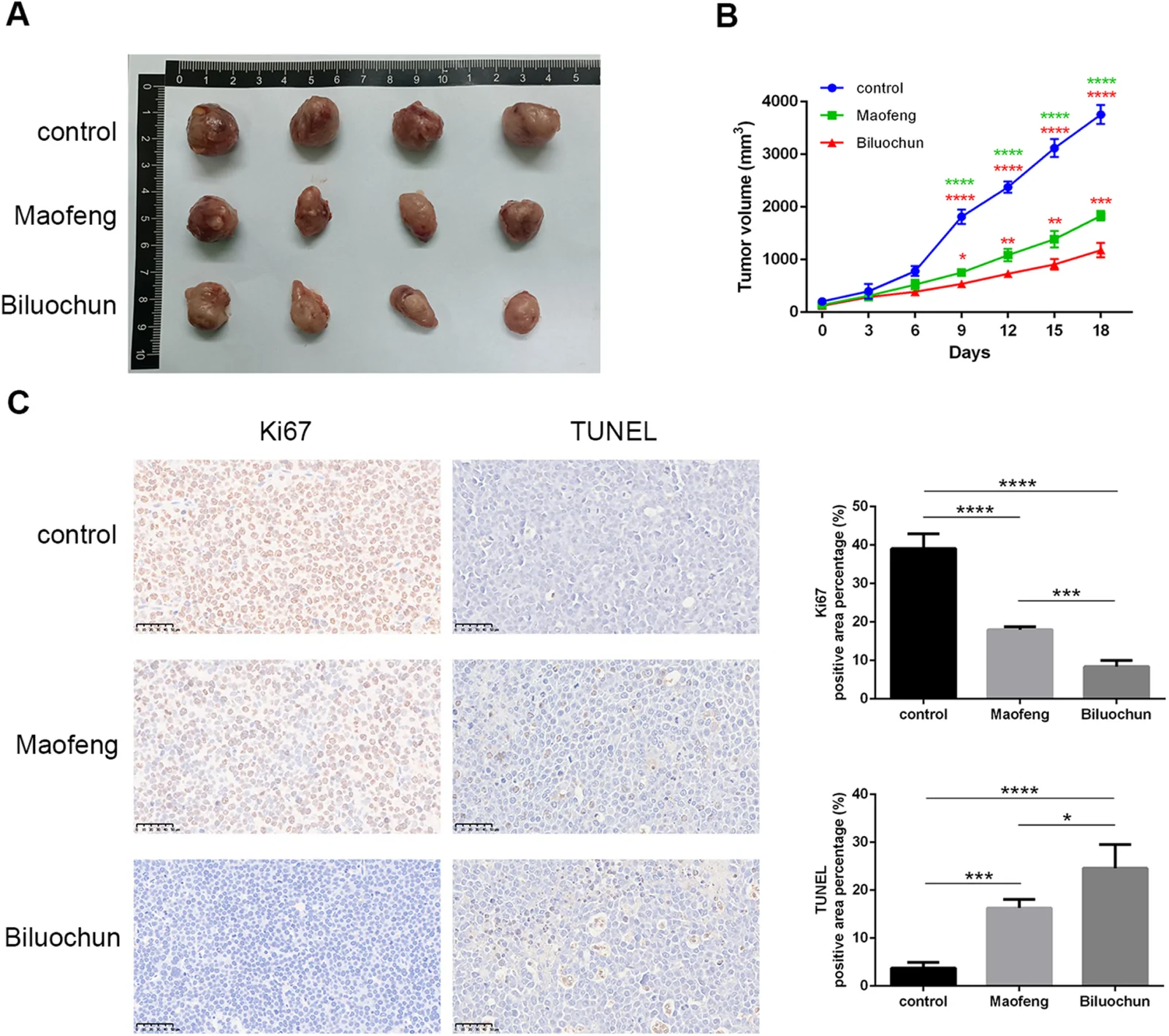
Antineoplastic impact of different tea decoctions on multiple myeloma (MM)-bearing mice. (A) When the tumor diameter reached the end point in the control group, three groups of mice were sacrificed simultaneously. Representative images of isolated tumor tissues from the control, Maofeng-treated, and Biluochun-treated groups were collected for visual morphological comparison. (B) After successful tumor formation, tumor diameter was measured with Vernier calipers every 3 days throughout the treatment period, and the corresponding tumor volumes were calculated to generate continuous tumor growth curves. (C) Immunohistochemical staining was performed on paraffin-embedded tumor tissue sections from the control, Maofeng, and Biluochun groups. Brown coloration in tissue sections indicates positive staining. The percentage of positive area relative to the total tissue area of each section was quantitatively analyzed using ImageJ software to comprehensively evaluate tumor proliferation and apoptosis. *P < 0.05, ***P < 0.001, ****P < 0.0001.

To elucidate the anti-myeloma efficacy of Biluochun infusion *in vivo*, an MM xenograft mouse model was established. Tumor-bearing mice were randomly divided into three groups and administered Biluochun tea infusion, Maofeng tea infusion, and an equal-volume of PBS vehicle solution as interventions. As shown in Figure 3A, statistical differences in tumor growth gradually became apparent among the groups. Starting from day 9 of treatment, both Biluochun and Maofeng infusions exerted remarkable suppressive effects on tumor growth *in vivo* compared with the control group. Notably, Biluochun infusion demonstrated more effective inhibition of tumor growth than Maofeng infusion and PBS (Fig. 3A). On day 18 of treatment, the control mice reached the study endpoint, and subsequently all mice in this experiment were sacrificed. Tumors from each mouse were completely excised and collected for macroscopic observation and comparative analysis. Consistent with the in vivo growth curve results, morphological observation showed that the Biluochun intervention group had the smallest tumor volume, while the PBS control group had the largest tumor mass and the Maofeng treatment group had an intermediate tumor size (Fig. 3B).

### Reduced Ki-67 and elevated TUNEL-positive cells in Biluochun infusion-treated MM tumor

Immunohistochemical staining for Ki-67 and TUNEL was conducted on paraffin-embedded tumor tissue sections derived from mice treated with Biluochun tea infusion, Maofeng tea infusion, or PBS. As shown in Figure 3C, compared with the PBS control group, both Biluochun and Maofeng infusions markedly decreased the proportion of Ki-67-positive proliferative cells and markedly increased the number of TUNEL-positive apoptotic cells in tumor tissue sections. Moreover, the Biluochun infusion exerted more potent biological effects, further reducing the proportion of Ki-67-positive cells and significantly increasing the number of TUNEL-positive cells compared with the Maofeng tea infusion. These findings indicate that both tea infusions inhibited tumor growth *in vivo* by inhibiting MM cell proliferation and inducing MM cell apoptosis, with the Biluochun tea infusion exhibiting superior efficacy in blocking proliferation and inducing apoptosis.

### Different components in four types of green tea infusions

**Figure 4.**
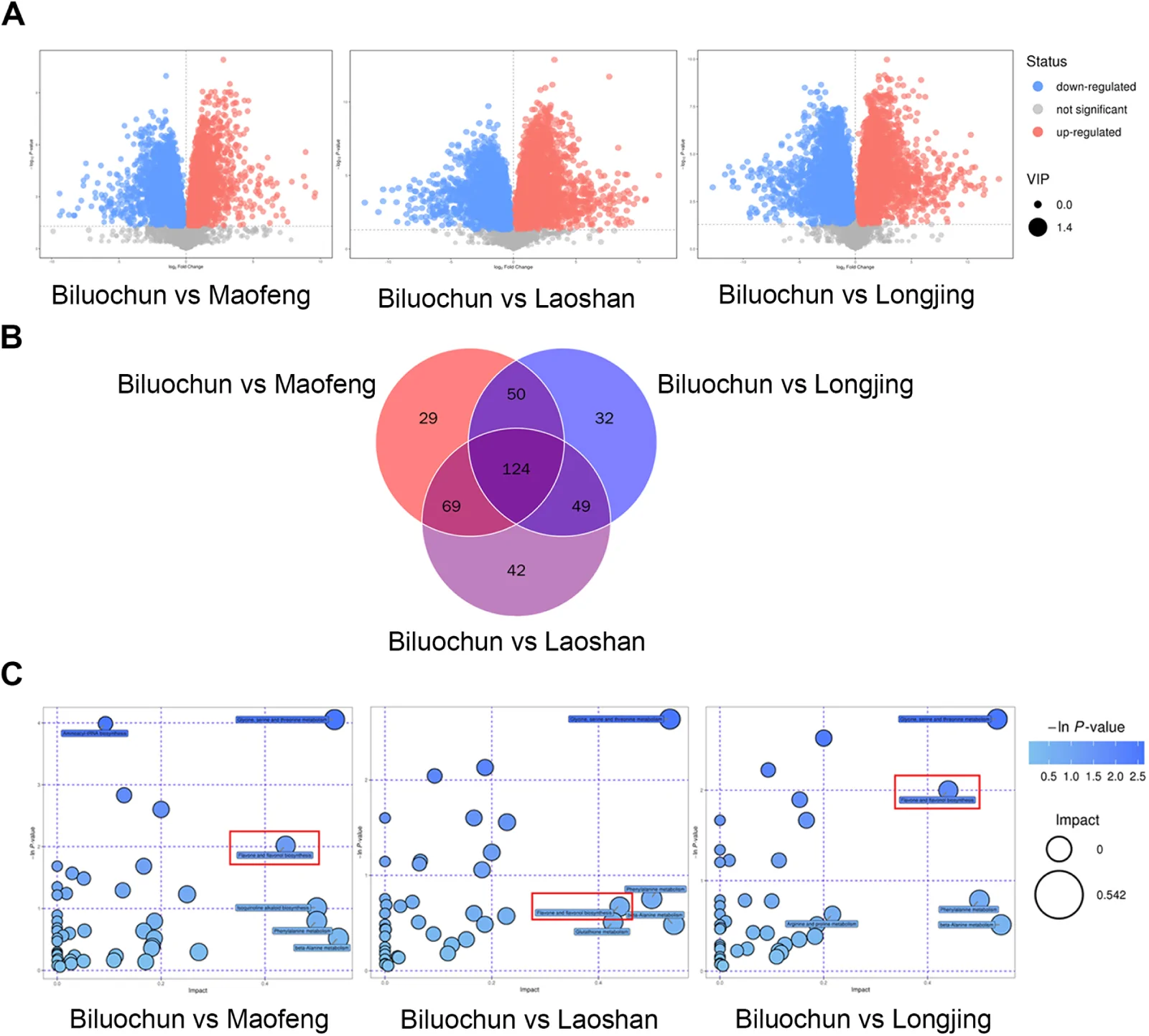
Mass spectrometry analysis of differential metabolites in four green tea decoctions. (A) Volcano plots for pairwise comparisons of metabolite profiles between Biluochun and the other three green teas. Each point in the figure represents a metabolite, the abscissa represents the fold change between groups for each metabolite (base-2 logarithm), the ordinate represents the *P*-value from Student’s *t*-test (the negative base-10 logarithm was used), and the point size represents the VIP score (variable importance in projection of the metabolite in the Orthogonal Projections to Latent structures discriminant analysis (OPLS-DA) model for this comparison. Significantly upregulated metabolites are indicated in red, significantly downregulated metabolites are indicated in blue, and non-significant metabolites are shown in gray. (B) Venn diagram showing the number of unique and overlapping differential metabolites from each pairwise comparison between Biluochun and the other three green tea groups. (C) Bubble plots of metabolic pathway enrichment analysis based on differential metabolites. Each bubble in the bubble plot represents a metabolic pathway, and the abscissa and size of the bubble represent the impact factor of the pathway in the topological analysis. The ordinate and color of the bubble represent the *P*-value of the enrichment analysis (the negative natural logarithm, −ln(*P*), was used); a darker color indicates a smaller *P*-value and more significant enrichment.

To identify potentially effective components contributing to the superior anti-MM activity of Biluochun tea infusion, a systematic mass spectrometry-based chemical composition analysis was performed. As illustrated in the volcano plot, each of the four tea infusions, when compared with Biluochun tea, exhibited differential upregulation (depicted in red) or downregulation (depicted in blue) of metabolites (Fig. 4A). To identify overlapping differential substances, a Venn diagram was constructed, revealing that a total of 124 common metabolites exhibited consistently significant differences in Biluochun tea when compared with each of the other three tea varieties (Fig. 4B). According to the Venn diagram and the differential metabolite table, we selected the substances in the three groups that simultaneously met the following conditions: (1) MS2 score (the score of secondary mass spectrometry spectral matching, ranging from 0 to 1, where a higher value is favorable) > 0.9; (2) *P*-value < 0.05; (3) the same trend across the three comparative groups (Table 1). In combination with the −ln *P*-value and pathway impact values presented in the enrichment bubble plot, pathway analysis revealed notable changes in glycine, serine, and threonine metabolism, as well as flavone and flavanol biosynthesis (Fig. 4C). Consequently, based on the differential metabolite table and pathway analysis, our focus was directed toward the differential metabolite DMY, involved in flavone and flavanol biosynthesis, as the target metabolite for subsequent experiments.

**Table 1:** Common differentially expressed metabolites of conditional screening.

MS2 name	MS2 score	FOLD CHANGE		
		Maofeng vs. Biluochun	Longjing vs. Biluochun	Laoshan vs. Biluochun
Trehalose	0.9931	0.2892	0.2315	0.4014
Traumatic Acid	0.9915	0.5528	0.5932	0.2078
Dihydromyricetin	0.9896	0.658	0.6314	0.5598
Isovitexin	0.9892	0.3722	0.3552	0.4882
Catechin	0.9879	0.8786	0.6741	0.8794
DL-Tyrosine	0.9769	0.8541	0.7654	0.8118
Ethyl caffeate	0.9683	0.7114	0.5698	0.5333
Mulberrofuran A	0.9589	0.5011	0.5996	0.5527
myo-Inositol	0.9364	0.8558	0.8044	0.8259

Conditional screening: (1) MS2 score > 0.9; (2) *P* < 0.05; (3) There was the same change trend in the three groups.

### DMY induced MM cell apoptosis through both mitochondrial and non-mitochondrial pathways

**Figure 5.**
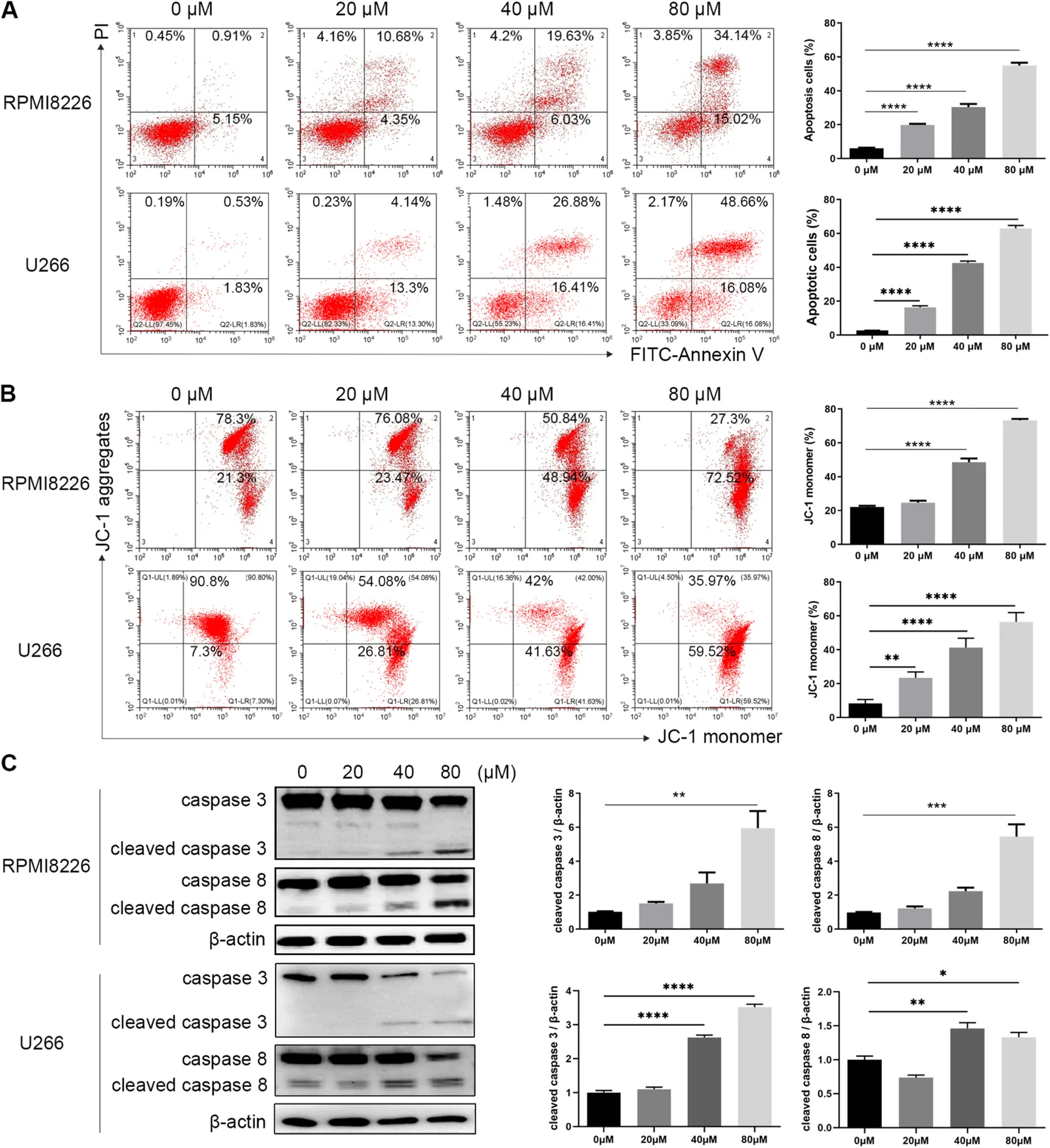
Apoptotic effects, changes in mitochondrial membrane potential, and expression of apoptosis-related proteins in RPMI8226 and U266 cells treated with DMY. (A) Flow cytometric analysis of the percentage of apoptotic RPMI8226 and U266 cells treated with different concentrations (0, 20, 40, and 80 μM) of DMY for 24 h. The 0 μM DMY group corresponds to the DMSO vehicle control. (B) RPMI8226 and U266 cells were treated with different concentrations (0, 20, 40, and 80 μM) of DMY for 24 h, and changes in mitochondrial membrane potential were examined via staining with the JC-1 working solution and analyzed via flow cytometry. (C) After treatment of RPMI8226 and U266 cells with different concentrations (0, 20, 40, and 80 μM) of DMY for 24 h, the expression of total and cleaved caspase-3 and caspase-8 was detected via western blot analysis. **P < 0.01, ***P < 0.001, ****P < 0.0001.

To further investigate the pro-apoptotic effects of DMY on MM, RPMI8226 and U266 cells were treated with graded concentrations (0, 20, 40, and 80 μM) of DMY for 24 h. Annexin V-FITC/PI double-staining was then performed to quantitatively assess the apoptotic rate in both cell lines. Flow cytometry results showed that DMY induced apoptosis in both RPMI8226 and U266 cells in a concentration-dependent manner (Fig. 5A). Meanwhile, the proportion of JC-1 monomer-positive cells in both RPMI8226 and U266 cells also increased with increasing DMY concentrations (Fig. 5B). Western blotting results demonstrated that the levels of cleaved caspase-3 and caspase-8 in both RPMI8226 and U266 cells gradually increased with increasing DMY concentrations (0, 20, 40, and 80 μM), significantly surpassing the control (Fig. 5C). These data suggest that DMY induces apoptosis of RPMI8226 and U266 cells through both mitochondrial and non-mitochondrial pathways.

## Discussion

Antitumor properties of green tea have been acknowledged and are largely attributed to EGCG. This predominant bioactive component possesses strong antioxidant activity^[^12^]^. Existing studies have demonstrated that EGCG can modulate the cell cycle^[^13^]^ and DNA methyltransferases ^[^14^]^. It also exerts suppressive effects on the mitogen-activated protein kinase (MAPK) and receptor tyrosine kinase pathways^[^15^]^. Apart from EGCG, multiple endogenous chemical constituents of green tea, including amino acids, caffeine, and soluble sugars, jointly participate in biological regulation, collectively endowing green tea with diverse pharmacological effects^[^16^]^. MM is a plasma cell malignancy that predominantly affects the elderly population. Preclinical exploration of green tea and its components against MM may provide candidate compounds for future therapeutic research. Nevertheless, relevant basic and clinical research remains insufficient at present. Additionally, green tea has a centuries-long history of cultivation across various regions in China. Variations in growing environments lead to distinct biological characteristics and chemical compositions among different green tea varieties. Identifying more favorable green tea varieties and understanding their corresponding chemical components hold significant potential for enhancing the treatment of MM, developing novel drugs, and offering guidance for a healthier lifestyle among elderly individuals.

In fact, numerous studies have demonstrated that tea quality and bioactive metabolite profiles are profoundly shaped by multiple terroir-dependent environmental factors, including regional topography, geomorphology, soil microorganisms^[^17^]^, soil physical and chemical conditions^[^18^]^, and elemental content, all of which collectively determine the differential biological activities of tea products. In the current study, to eliminate the confounding effects of atmospheric pollution and to standardize external environmental variables, four representative Chinese mountain green teas with distinct geographical characteristics were carefully selected: Maofeng from Mount Emei in Sichuan Province, Biluochun from Dongting Mountain in Jiangsu Province, Laoshan Green Tea from Qingdao in Shandong Province, and Longjing from Hangzhou in Zhejiang Province. Moreover, these teas are cultivated in diverse areas characterized by varying topographies and landforms, including slopes, hills and mountains, basins, and plateau junctions. They are grown in different types of soils, such as acidic^[^18^]^, natural yellow-brown soils, soils developed from granite parent rock, and ordinary brown soils rich in organic matter^[^19^]^. Such significant differences in growth conditions provide a natural experimental basis for comparing the chemical components and differences in anti-myeloma biological activity among various green tea varieties.

Our novel findings revealed that aqueous extracts derived from the four green teas significantly induced apoptosis in MM cells (RPMI8226 and U266 cell line) in a concentration-dependent manner. Notably, Biluochun infusion treatment demonstrated the strongest suppressive capacity against tumor proliferation in vivo animal assays. Additionally, Biluochun treatment achieved more effective restraint on tumor cell proliferation and markedly facilitated apoptotic progression inside tumor tissues, compared with other infusions and the control group. Although the tea component EGCG has been widely studied for its anticancer effect, according to our mass spectrometry analysis, EGCG did not serve as the shared differential metabolite accounting for the functional discrepancies among the four tea groups. Therefore, we did not consider this component as the main object of analysis. As expected, metabolomic profiling via mass spectrometry detection revealed a greater abundance of DMY in the Biluochun infusion compared to the other three infusions. Combined with screening results of shared differential metabolites and metabolic pathway enrichment analysis, we chose DMY as the main common differential metabolite for the following study. We preliminarily speculate that the differing anti-myeloma activity across various tea infusions may be partially linked to their distinct dihydromyricetin levels, which requires further validation. DMY, also known as ampelopsin, is a natural flavonoid widely present in various plant tissues. Studies have shown that DMY exhibits favorable therapeutic effects against multiple solid tumors such as lung cancer, breast cancer and osteosarcoma^[^20–22^]^. Its antitumor bioactivities are shown to be mediated via multiple signaling cascades, including NF-κB-Nrf2, AMPK and/or MAPK pathways, as well as the activation of endoplasmic reticulum stress^[^23–25^]^. However, there are no relevant studies on its therapeutic effect on hematological tumors. Our results showed that DMY significantly induced MM cell apoptosis and impeded mitochondrial membrane potential of MM cells in a concentration dependent manner, suggesting involvement of mitochondrial mediated apoptosis pathway. It is well known that caspases are a family of protease enzymes that serve as core regulators governing the apoptotic process, which were generally categorized into two major subgroups, namely initiator caspases represented by caspase-8 and caspase-9, and effector caspases represented by caspase-3 and caspase-7. As a pivotal executioner caspase, Caspase-3 was originally named cysteine protease protein 32 kD, because it encodes a 32 kD cysteine aspartate-specific protease 9^[^26^]^. Caspase-3 is typically activated through interactions with upstream caspases, such as caspase-8 or caspase-9. Upon activation, caspase-3 undergoes proteolytic cleavage and transforms into its biologically active form. The activated caspase-3 further targets and cleaves diverse intracellular structural and functional proteins, ultimately driving the progression of cellular apoptosis^[^27,28^]^. Caspase-8 is a cysteine-aspartate-specific protease that commonly initiates exogenous apoptotic pathways upon the activation of cell surface death receptors^[^29^]^. In this study, Western blot detection revealed that the expression levels of cleaved caspase-3 and caspase-8 were markedly increased in RPMI8226 and U266 cells treated by DMY. Collectively, these findings illustrate that DMY induces myeloma cell apoptosis through the combined activation of intrinsic mitochondrial pathway and extrinsic non-mitochondrial pathway.

Several limitations of the present study should be noted for further investigation. Although we identified DMY as a key characteristic component of Biluochun tea and verified its anti-myeloma effects through in vitro and in vivo experiments, the specific direct molecular target of DMY remains to be further explored. In addition, this study focused on the intrinsic apoptotic mechanism, and the potential impact of DMY on the tumor microenvironment requires more in-depth evaluation. Additionally, systematic pharmacokinetic validation of the oral bioavailability and tissue distribution of DMY following tea intake is lacking, and combinatorial treatments with standard clinical myeloma chemotherapeutics to fully verify synergistic adjuvant therapeutic effects were not assessed. There is also a lack of experimental evidence supporting tumor-preventive properties of Biluochun tea or DMY, as the present results only demonstrate anti-proliferative and pro-apoptotic activity against already formed myeloma tumor cells. Furthermore, as a preliminary preclinical study, the optimal administration strategy and pharmacokinetic properties of DMY still need to be optimized in future research, and clinical translation will require more systematic validation.

In summary, this work provides a novel research perspective for exploring the anti-MM activity of Biluochun infusion and its constituent, DMY, by triggering both the mitochondrial and extrinsic apoptosis pathways. This preclinical research preliminarily demonstrates the anti-myeloma phenotype of Biluochun tea and its enriched component, DMY, in cell and xenograft models, offering a research foundation for subsequent mechanistic and translational exploration. The identification of the active ingredient, DMY, may pave the way for new targeted therapeutic agents against MM following layered follow-up research.

## Data Availability

The datasets analyzed and/or used during the present study are obtainable from the corresponding author upon reasonable request.

## Research registration unique identifying number (UIN)

This study does not require Research Registration Unique Identifying Number, as it does not involve patient experimentation.

## References

[1] Li H Kong D Zhao Y. Irisin protected hemopoietic stem cells and improved outcome of severe bone marrow failure. Biomed Pharmacother 2023;169:115863. 10.1016/j.biopha.2023.115863 37952356

[2] Cowan AJ Green DJ Kwok M. Diagnosis and management of multiple myeloma: a review. Jama 2022;327:464–77. 10.1001/jama.2022.0003 35103762

[3] van de Donk N Pawlyn C Yong KL. Multiple myeloma. Lancet 2021;397:410–27. 10.1016/S0140-6736(21)00135-5 33516340

[4] Luo Q Luo L Zhao J. Biological potential and mechanisms of Tea’s bioactive compounds: an updated review. J Adv Res 2024;65:345–63. 10.1016/j.jare.2023.12.004 PMC11519742 38056775

[5] Yan Z Zhong Y Duan Y. Antioxidant mechanism of tea polyphenols and its impact on health benefits. Anim Nutr 2020;6:115–23. 10.1016/j.aninu.2020.01.001 PMC7283370 32542190

[6] Schramm L. Going Green. the role of the green tea component EGCG in chemoprevention. J Carcinog Mutagen 2013;4:1000142. 10.4172/2157-2518.1000142 PMC3783360 24077764

[7] Gan RY Li HB Sui ZQ. Absorption, metabolism, anti-cancer effect and molecular targets of epigallocatechin gallate (EGCG): an updated review. Crit Rev Food Sci 2018;58:924–41. 10.1080/10408398.2016.1231168 27645804

[8] Zhang X Han Y Fan C. Epigallocatechin gallate induces apoptosis in multiple myeloma cells through endoplasmic reticulum stress induction and cytoskeletal disruption. Int Immunopharmacol 2024;141:112950. 10.1016/j.intimp.2024.112950 39159563

[9] Agha RA Mathew G Rashid R. Transparency in the reporting of artificial intelligence – the TITAN guideline. Prem J Sci 2025;10:100082.

[10] Ren J Feng X Guo Y. GSK-3β/β-catenin pathway plays crucial roles in the regulation of NK cell cytotoxicity against myeloma cells. FASEB J 2023;37:e22821. 10.1096/fj.202201658RR 36794671

[11] Kilkenny C Browne WJ Cuthill IC. Improving bioscience research reporting: the ARRIVE guidelines for reporting animal research. PLoS Biol 2010;8:e1000412. 10.1371/journal.pbio.1000412 PMC2893951 20613859

[12] Lambert JD Elias RJ. The antioxidant and pro-oxidant activities of green tea polyphenols: a role in cancer prevention. Arch Biochem Biophys 2010;501:65–72. 10.1016/j.abb.2010.06.013 PMC2946098 20558130

[13] Sasaki K Fujita K Fujihara S. The Polyphenol (-)-Epigallocatechin-3-gallate (EGCG) Inhibits the Proliferation of Gastric Cancer Cells and Alters microRNA Signatures. Anticancer Res 2025;45:2925–36. 10.21873/anticanres.17660 40578936

[14] Lee AH Fraser ML Binns CW. Possible role for green tea in ovarian cancer prevention. Future Oncol 2005;1:771–77. 10.2217/14796694.1.6.771 16556056

[15] Singh BN Shankar S Srivastava RK. Green tea catechin, epigallocatechin-3-gallate (EGCG): mechanisms, perspectives and clinical applications. Biochem Pharmacol 2011;82:1807–21. 10.1016/j.bcp.2011.07.093 PMC4082721 21827739

[16] Rameshrad M Razavi BM Hosseinzadeh H. Protective effects of green tea and its main constituents against natural and chemical toxins: a comprehensive review. Food Chem Toxicol 2017;100:115–37. 10.1016/j.fct.2016.11.035 27915048

[17] Bag S Mondal A Banik A. Exploring tea (Camellia sinensis) microbiome: insights into the functional characteristics and their impact on tea growth promotion. Microbiol Res 2022;254:126890. 10.1016/j.micres.2021.126890 34689100

[18] Ding ZJ Shi YZ Li GX. Tease out the future: how tea research might enable crop breeding for acid soil tolerance. Plant Commun 2021;2:100182. 10.1016/j.xplc.2021.100182 PMC8132122 34027395

[19] Xiang J Rao S Chen Q. Research progress on the effects of selenium on the growth and quality of tea plants. Plants-Basel 2022;11:2491. 10.3390/plants11192491 PMC9573726 36235356

[20] Fan KJ Yang B Liu Y. Inhibition of human lung cancer proliferation through targeting stromal fibroblasts by dihydromyricetin. Mol Med Rep 2017;16:9758–62. 10.3892/mmr.2017.7802 29039563

[21] Li Y Zhou Y Wang M. Ampelopsin inhibits breast cancer cell growth through mitochondrial apoptosis pathway. Biol Pharm Bull 2021;44:1738–45. 10.1248/bpb.b21-00470 34470980

[22] Wang Y Wang W Qiu E. Protection of oxidative stress induced apoptosis in osteosarcoma cells Saudi journal of biological sciences by dihydromyricetin through down-regulation of caspase activation and up-regulation of BcL-2. Saudi J Biol Sci 2017;24:837–42. 10.1016/j.sjbs.2016.12.004 PMC5415116 28490955

[23] Park GB Jeong JY Kim D. Ampelopsin-induced reactive oxygen species enhance the apoptosis of colon cancer cells by activating endoplasmic reticulum stress-mediated AMPK/MAPK/XAF1 signaling. Oncol Lett 2017;14:7947–56. 10.3892/ol.2017.7255 PMC5727598 29250183

[24] Wang Z Sun X Feng Y. Dihydromyricetin reverses MRP2-induced multidrug resistance by preventing NF-κB-Nrf2 signaling in colorectal cancer cell. Phytomedicine 2021;82:153414. 10.1016/j.phymed.2020.153414 33461143

[25] Wang Z Sun X Feng Y. Dihydromyricetin reverses MRP2-mediated MDR and enhances anticancer activity induced by oxaliplatin in colorectal cancer cells. Anti-cancer Drug 2017;28:281–88. 10.1097/CAD.0000000000000459 27997436

[26] Jiang M Qi L Li L. The caspase-3/GSDME signal pathway as a switch between apoptosis and pyroptosis in cancer. Cell Death Discov 2020;6:112. 10.1038/s41420-020-00349-0 PMC7595122 33133646

[27] McIlwain DR Berger T Mak TW. Caspase functions in cell death and disease. Csh Perspect Biol 2015;7:a026716. 10.1101/cshperspect.a026716 PMC4382736 25833847

[28] Huang J Wu L Zhao Y. Programmed cell death of chondrocytes, synovial cells, osteoclasts, and subchondral bone cells in osteoarthritis. J Inflamm Res 2025;18:12323–60. 10.2147/JIR.S514309 PMC12428662 40951445

[29] Mandal R Barrón JC Kostova I. Caspase-8: the double-edged sword. Bba-Rev Cancer 2020;1873:188357. 10.1016/j.bbcan.2020.188357 32147543

